# Machine learning models of clinically relevant biomarkers for the prediction of stable obstructive coronary artery disease

**DOI:** 10.3389/fcvm.2022.933803

**Published:** 2022-07-19

**Authors:** Juntae Kim, Su Yeon Lee, Byung Hee Cha, Wonseop Lee, JiWung Ryu, Young Hak Chung, Dongmin Kim, Seong-Hoon Lim, Tae Soo Kang, Byoung-Eun Park, Myung-Yong Lee, Sungsoo Cho

**Affiliations:** ^1^Division of Cardiovascular Medicine, Department of Internal Medicine, Dankook University Hospital, Dankook University College of Medicine, Cheonan-si, South Korea; ^2^CNAI, Seoul, South Korea; ^3^Department of Cardiology, Heart and Brain Hospital, Chung-Ang University Gwangmyeong Hospital, Chung-Ang University College of Medicine, Gwangmyeong, South Korea

**Keywords:** machine learning, artificial intelligence, coronary artery disease, stable angina pectoris, personalized medicine

## Abstract

**Background:**

In patients with suspected obstructive coronary artery disease (CAD), evaluation using a pre-test probability model is the key element for diagnosis; however, its accuracy is controversial. This study aimed to develop machine learning (ML) models using clinically relevant biomarkers to predict the presence of stable obstructive CAD and to compare ML models with an established pre-test probability of CAD models.

**Methods:**

Eight machine learning models for prediction of obstructive CAD were trained on a cohort of 1,312 patients [randomly split into the training (80%) and internal validation sets (20%)]. Twelve clinical and blood biomarker features assessed on admission were used to inform the models. We compared the best-performing ML model and established the pre-test probability of CAD (updated Diamond-Forrester and CAD consortium) models.

**Results:**

The CatBoost algorithm model showed the best performance (area under the receiver operating characteristics, AUROC, 0.796, and 95% confidence interval, CI, 0.740–0.853; Matthews correlation coefficient, MCC, 0.448) compared to the seven other algorithms. The CatBoost algorithm model improved risk prediction compared with the CAD consortium clinical model (AUROC 0.727; 95% CI 0.664–0.789; MCC 0.313). The accuracy of the ML model was 74.6%. Age, sex, hypertension, high-sensitivity cardiac troponin T, hemoglobin A1c, triglyceride, and high-density lipoprotein cholesterol levels contributed most to obstructive CAD prediction.

**Conclusion:**

The ML models using clinically relevant biomarkers provided high accuracy for stable obstructive CAD prediction. In real-world practice, employing such an approach could improve discrimination of patients with suspected obstructive CAD and help select appropriate non-invasive testing for ischemia.

## Introduction

Estimating the probability of coronary artery disease (CAD) in patients with stable angina or anginal equivalent symptoms is a frequent challenge. The current guidelines recommend estimation of the pre-test probability of CAD scores to guide decisions on whether diagnostic testing could be deferred or performed, and whether the initial test should be non-invasive or invasive (1). However, recent studies have shown that the performance of the traditional pre-test probability of CAD models is limited in estimation of obstructive CAD (2, 3). Moreover, the pre-test probability of CAD models does not reflect the current regulatory status of risk factors such as hypertension, diabetes mellitus (DM), and dyslipidemia.

Machine learning (ML) involves the application of artificial intelligence (AI) that uses computer algorithms to identify patterns in large datasets with a multitude of variables to capture high-dimensional, non-linear relationships among clinical features. Data-driven techniques based on ML can improve the performance of risk predictions by exploiting large data repositories to identify novel risk predictors agnostically and more complex interactions between them. However, only few studies have been conducted on stable obstructive CAD using ML of clinical risk factors and blood biomarkers commonly used in clinical practice. Therefore, we aimed to develop ML models using these features to predict stable obstructive CAD and determine the ranking of the features’ predictive contribution. We also compared the ML models with the established pre-test probability of CAD models to evaluate whether there were significant improvements in discrimination.

## Method

### Study population

We included a cohort of 4,906 patients who visited the outpatient department for angina or anginal equivalent symptoms and underwent invasive coronary angiography at Dankook University Hospital between August 2014 and January 2016. Obstructive CAD was defined as any stenosis 70% or greater in the epicardial coronary artery, 50% or greater in the left main coronary artery, or both. Non-obstructive CAD was defined as a stenosis 20% or greater but less than 70% in any other epicardial coronary artery, or a coronary artery stenosis 20% or greater but less than 50% in the left main coronary artery, as recorded by physicians in the catheterization report. No apparent CAD was defined as all coronary stenoses less than 20% or luminal irregularities. The case group was defined as having obstructive CAD, and the control group was defined as having no apparent CAD. When creating ML models, the inclusion criteria were patients who were diagnosed with chronic stable coronary syndrome after visiting the outpatient department with angina or anginal equivalent symptoms; the exclusion criteria were patients who were diagnosed with acute myocardial infarction (AMI) based on the fifth universal definition of myocardial infarction, had non-obstructive moderate CAD (20–70% stenosis), and previously underwent percutaneous coronary intervention (PCI).

**FIGURE 1:**
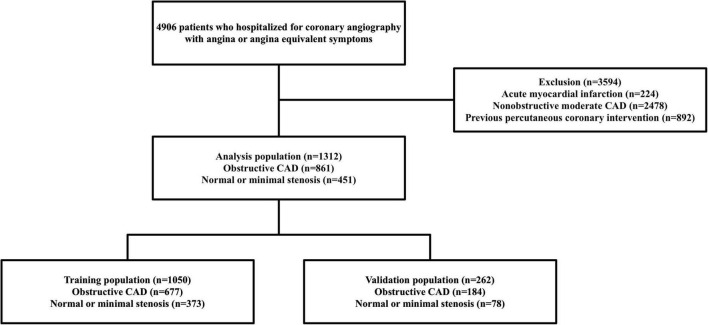
Flowchart of the study population and process. CAD, coronary artery disease.

Finally, 1,312 patients (case group = 861, control group = 451) were selected for the analysis. A subset of the dataset was randomly selected to train the risk-prediction algorithms, and the remaining dataset was used for validation (Figure 1). This study was approved by the Institutional Review Board of the Dankook University Hospital (2018-09-014).

### Data collection

Baseline information was collected from patients with suspected CAD admitted for invasive coronary angiography, including demographics, cardiovascular risk factors [hypertension, DM, dyslipidemia, chronic kidney disease (CKD), and smoking status], and biomarkers [hemoglobin A1c (HbA1c), creatinine clearance, high-sensitivity cardiac troponin T (troponin T), and lipid profile]. These parameters were also used in the established pre-test probability scores for analysis.

### Machine learning algorithms and feature importance

Eight supervised ML algorithms were selected: CatBoost (4), Extreme gradient (XG) boost (5), gradient boost (6), Light Gradient Boosting Machine (lightGBM) (7), MultiLayer Perceptron (MLP) (8), support vector machine with a linear kernel (SVM) (9), Random forest (10), and K-nearest neighbor (11). Each ML model was implemented using Python 3.8.2, with the following packages: *xgboost* for extreme gradient boost, *catboost* for CatBoost, *lightgbm* for lightGBM, *pytorch* for MultiLayer Perceptron, and *scikit-learn* for the other ML algorithms. For the MLP and SVM algorithms, categorical features were represented by one-hot encoding. Hyperparameters were tuned using the Bayesian hyperparameter tuning library *optuna* with fivefold cross-validation on the training population (Supplementary Table 1). To interpret the ML prediction models, we used SHapley Additive exPlanations (SHAP). The SHAP value assesses the impact of each variable by representing the change in log odds when a variable is hidden from the model (12). The MissForest algorithm was used for imputation of missing values in the ML models, except for boosting algorithms (13).

The study population was randomly split into the training (80%; case group = 677, control group 373) and validation (20%; case group = 184, control group = 78) sets. To control the overfitting caused by an imbalanced dataset, the bootstrap resampling method was applied, obtaining equal proportions of numbers in each group of the training population (10 bootstrap samples: case group = 373, control group = 373). To evaluate feature importance, we estimated the SHAP values of 48 available variables in the CatBoost model (Supplementary Figure 1). Twelve variables for obstructive CAD were selected in the final prediction models based on the recursive feature elimination and visual inspection of a SHAP-dependence plot.

### Statistical analysis

The Revised Diamond-Forrester score (2), CAD consortium basic, and CAD consortium clinical (14) were calculated to compare model performance. The models were compared with ML-based models by the area under the receiver operating characteristics (AUROC) using the DeLong method (15) and Matthews correlation coefficient (MCC) (16). The MCC is a useful metric for evaluating binary classification, especially for imbalanced datasets. Continuous variables were expressed as mean ± standard deviation (SD) or median (interquartile range) and were compared by Student’s *t*-tests or Wilcoxon rank-sum tests. Categorical variables were expressed as proportions and compared by χ^2^ test. A two-sided *p*-value < 0.05 was considered significant for all the analyses.

## Results

### Patient characteristics

Table 1 presents the baseline characteristics of the development and validation datasets. The mean age of the 1,312 patients was 63 ± 11.8 years, and 59.4% were men. The CAD group was significantly older, had higher systolic blood pressure, and more frequent hypertension, DM, and dyslipidemia than the no CAD group. Moreover, the CAD group had higher levels of HbA1c, troponin T, and triglycerides than the no CAD group. In contrast, creatinine clearance and high-density lipoprotein (HDL) cholesterol levels were significantly lower in the case group.

**TABLE 1:** Baseline characteristics.

Features	Total population (*n* = 1312)	Control group (*n* = 451)	Case group (*n* = 861)	*p*-value
Age (years)	63.0 ± 11.8	58.6 ± 12.4	65.6 ± 10.7	< 0.001
Male gender	806 (59.4%)	240 (48.4%)	566 (65.7%)	< 0.001
Hypertension	1003 (73.9%)	310 (62.5%)	693 (80.4%)	< 0.001
Diabetes mellitus	572 (42.1%)	148 (29.8%)	424 (49.2%)	< 0.001
Dyslipidemia	1018 (77.6%)	367 (74.0%)	691 (80.2%)	0.010
Cerebrovascular accident	112 (8.2%)	37 (7.5%)	75 (8.7%)	0.485
Chronic kidney disease	63 (4.6%)	9 (1.8%)	54 (6.3%)	< 0.001
Smoking	542 (39.9%)	158 (31.9%)	384 (44.5%)	< 0.001
Non-smoking	816 (60.1%)	338 (68.1%)	478 (55.5%)	
Current-smoking	248 (18.3%)	68 (13.7%)	180 (20.9%)	
Ex-smoker	294 (21.6%)	90 (18.1%)	204 (23.7%)	
BMI (kg/m^2^)	25.1 ± 3.5	25.2 ± 3.7	25.0 ± 3.4	0.304
Systolic blood pressure (mmHg)	135.2 ± 19.6	132.3 ± 18.7	136.9 ± 20.0	< 0.001
Diastolic blood pressure (mmHg)	81.4 ± 13.5	82.0 ± 13.6	81.1 ± 13.4	0.257
Hemoglobin (g/dL)	13.4 (12.3, 14.5)	13.3 (12.4, 14.3)	13.5 (12.1, 14.5)	0.609
Hematocrit (%)	39.7 (36.5, 42.5)	39.6 (37.0, 42.2)	39.7 (36.1, 42.7)	0.570
Creatinine clearance (mL/min/1.73 m^2^)	79.9 (60.2, 101.3)	88.8 (68.9, 112.7)	75.8 (54.3, 95.9)	< 0.001
Total cholesterol (mg/dL)	159.0 (135.0, 189.0)	159.0 (137.0, 188.5)	159.0 (134.0, 189.0)	0.643
LDL cholesterol (mg/dL)	88.0 (65.2, 116.2)	90.6 (66.8, 116.0)	87.0 (64.5, 116.4)	0.702
HDL cholesterol (mg/dL)	42.0 (35.0, 51.0(	46.0 (37.0, 56.0)	40.0 (34.0, 48.0)	< 0.001
Triglyceride (mg/dL)	130.0 (88.0, 190.0)	115.0 (77.8, 177.2)	136.5 (95.2, 200.0)	< 0.001
Glucose (mg/dL)	120.5 (103.0, 154.0)	112.0 (102.0, 135.8)	126.5 (104.0, 162.8)	< 0.001
HbA1c (%)	5.9 (5.5, 6.5)	5.7 (5.5, 6.1)	6.0 (5.6, 6.8)	< 0.001
Troponin T (ng/mL)	0.010 (0.005, 0.020)	0.010 (0.003, 0.010)	0.010 (0.007, 0.021)	0.036
LDH (mg/dL)	201.0 (177.0, 242.2)	205.0 (179.5, 243.0)	200.0 (176.0, 242.0)	0.222
NT-proBNP (pg/mL)	89.9 (36.5, 526.2)	60.0 (25.1, 254.6)	117.7 (47.9, 711.8)	0.908

*Values are n (%), mean ± SD (standard deviation), or median (Q1, Q3). BMI, body mass index; CRP, C-reactive protein; HbA1c, hemoglobin A1c; HDL, high-density lipoprotein; LDH, lactate dehydrogenase; LDL, low-density lipoprotein; NT-proBNP, N-terminal pro-brain natriuretic peptide.*

### Model performance and comparison to the established model

**FIGURE 2:**
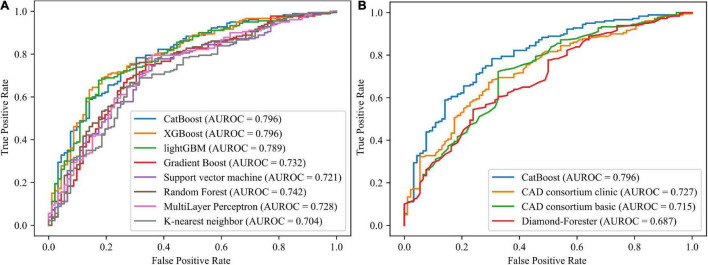
Receiver operating characteristic curves for the machine learning models and established pre-test probability of CAD models. **(A)** Comparing the eight machine learning models. **(B)** Comparing the CatBoost model and the established pre-test probability of CAD models. AUROC, area under the receiver operating characteristics; CAD, coronary artery disease; GBM, gradient boosting machine; XG, extreme gradient.

Using 12 potential variables, prediction models for stable obstructive CAD were developed with eight ML algorithms. Among the eight ML-based models, the highest predictive performance was observed for CatBoost (AUROC 0.796; 95% CI 0.74–0.853; MCC 0.448), performing similarly to XGboost (AUROC 0.796; 95% CI 0.74–0.852; MCC 0.399) and lightGBM (AUROC 0.789; 95% CI 0.732–0.846; MCC 0.403), slightly better than random forest (AUROC 0.742; 95% CI 0.679–0.805; MCC 0.413), gradient boost (AUROC 0.732; 95% CI 0.667–0.796; MCC 0.371), Multilayer Perceptron (AUROC 0.728; 95% CI 0.663–0.792; MCC 0.379), and support vector machine (AUROC 0.721; 95% CI 0.657–0.786; MCC 0.373), and significantly better than the K-nearest neighbor model (AUROC = 0.704; 95% CI 0.638–0.77; MCC 0.313) in the independent validation set (Figure 2A). The CatBoost model also performed significantly better than the established pre-test probability of CAD scores, the CAD consortium clinical model (AUROC 0.727; 95% CI 0.664–0.789; MCC 0.313) and Diamond-Forrester score (AUROC 0.687; 95% CI 0.621–0.753; MCC 0.271) (Figure 2B). The AUROC, MCC, accuracy, sensitivity, specificity, positive predictive value, negative predictive value, and F1 of all the risk prediction models are presented in Table 2.

**TABLE 2:** Comparison of performance between risk prediction models.

Models	AUROC	MCC	Accuracy	Sensitivity	Specificity	PPV	NPV	F1
CatBoost	**0.796**	**0.448**	**0.746**	0.783	0.674	**0.825**	0.614	0.803
XGBoost	**0.796**	0.399	0.724	0.767	0.641	0.807	0.584	0.786
LightGBM	0.789	0.403	0.724	0.761	0.652	0.811	0.583	0.785
Random forest	0.742	0.413	0.728	0.761	0.663	0.815	0.587	0.787
Gradient boost	0.732	0.371	0.710	0.750	0.630	0.799	0.563	0.774
Linear SVM	0.721	0.373	0.699	0.706	0.685	0.814	0.543	0.756
MLP	0.728	0.379	0.710	0.739	0.652	0.806	0.561	0.771
CAD consortium clinical	0.727	0.313	0.676	0.706	0.620	0.784	0.518	0.743
CAD consortium basic	0.715	0.223	0.559	0.444	**0.783**	0.800	0.419	0.571
Diamond-Forrester score	0.687	0.271	0.706	**0.933**	0.261	0.712	**0.667**	**0.808**
K-nearest neighbor	0.704	0.313	0.676	0.706	0.620	0.784	0.518	0.743

*AUROC, area under the receiver operating characteristics; CAD, coronary artery disease; GBM, gradient boosting machine; MCC, Matthews correlation coefficients; NPV, negative predictive value; PPV, positive predictive value; SVM, support vector machine; XG, extreme gradient. The bold values indicate the best performance of the 11 models.*

### Feature importance

**FIGURE 3:**
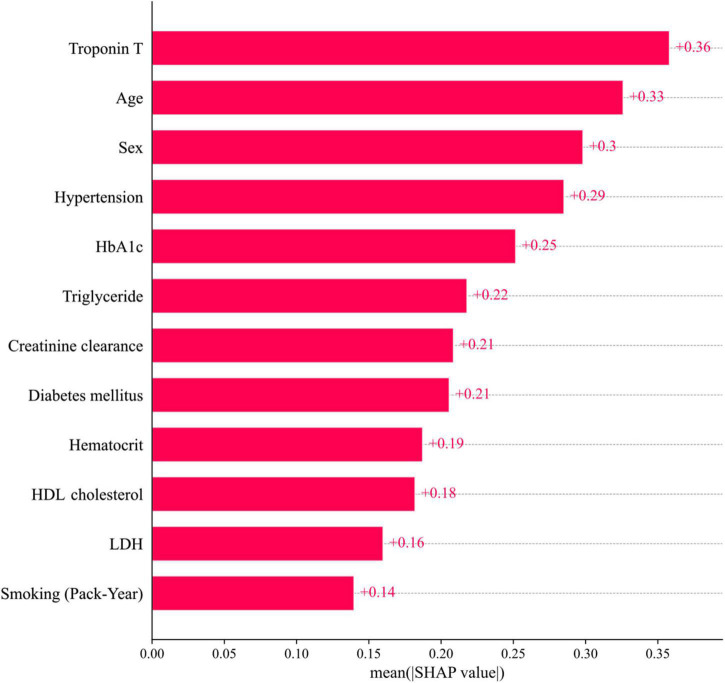
Feature importance ranking. **(A)** Mean SHAP value of features. **(B)** Impact on CatBoost model output of SHAP value. HbA1c, hemoglobin A1c; HDL, high-density lipoprotein; LDH, lactate dehydrogenase; SHAP, SHapley Additive exPlanations; Troponin T, high-sensitivity cardiac troponin T.

**FIGURE 4:**
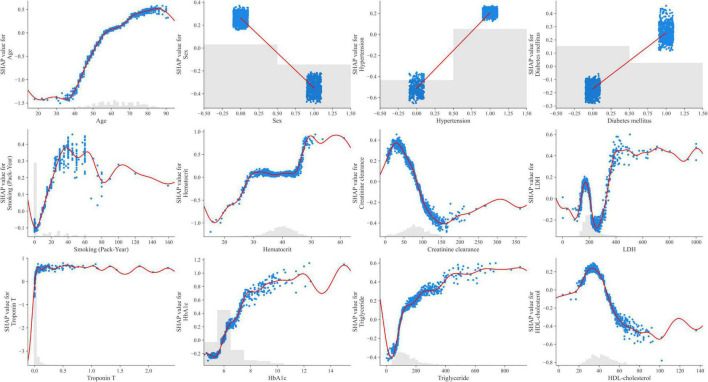
SHAP dependence plots of the CatBoost model. HbA1c, hemoglobin A1c; HDL, high-density lipoprotein; LDH, lactate dehydrogenase; SHAP, SHapley Additive exPlanations; Troponin T, high-sensitivity cardiac troponin T.

The 12 potential variables for stable obstructive CAD prediction were ranked using SHAP values. Age, sex, hypertension, troponin T, HbA1c, triglycerides, and HDL cholesterol were important features in our study (Figure 3A). To identify features that influenced the prediction model, we constructed a SHAP summary plot of CatBoost. The plot shows how the variable values are related to the SHAP values in the training dataset. Higher SHAP values were associated with higher CAD probability (Figure 3B). The SHAP-dependence plot (Figure 4) can also be used to understand how a single feature affects the output of the CatBoost prediction model. The *y*-axis values indicate the SHAP values of the features, and the values of features for the *x*-axis were in the SHAP-dependence plot. In the plot, we visualized how the influence of a feature changed as its values varied. SHAP values exceeding zero for specific features represent increased risk of CAD.

## Discussion

The main findings of our analysis were as follows: (1) the ML-based model (CatBoost), using clinically relevant biomarkers, exhibited a more accurate prediction of stable obstructive CAD than the established pre-test probability models, (2) using a novel ML-based model, we identified important features for the diagnosis of obstructive CAD.

Accurate prediction of obstructive stable CAD still represents an unmet need. Current guidelines recommend assessing the probability of obstructive CAD from clinical risk factors and, according to this pre-test, probability refers to non-invasive testing, invasive coronary angiography, or no further assessment (1). However, the diagnostic performance of established pre-test probability models is limited in the estimation of obstructive CAD in contemporary cohorts. Previous data have shown that the current model overestimates the probability of obstructive CAD in unselected patients (17). Another study demonstrated that the updated 2019 ESC guideline pre-test probability recommendations tended to underestimate slightly the disease in the SCOT-Heart trial cohort (18).

As the ML algorithm has been recently used for the diagnosis and prognosis of coronary artery disease, its predictive ability has improved significantly compared with established pre-test and prediction models. In the CREATION cohort study, the ML model provided better accuracy and discrimination than the existing traditional model. Using the ML method instead of established pre-test probability models (modified Diamond-Forrester and CAD consortium score) would imply a correct change in diagnostic strategy in 22.2% of the patients (19). From the CONFIRM registry, it has been shown that an ML model combining clinical features and coronary artery calcium score can accurately estimate the pre-test probability of CAD (20). Also, recent studies have attempted to diagnose stable CAD using multiple biomarkers, but there are limitations regarding difficulties in direct clinical practice application (21).

Only few studies have been conducted on stable obstructive CAD prediction by incorporating multiple biomarkers into the ML algorithm. The ML-based model could be more accurate and account for subtleties in data that are overlooked by linear assumption. In this study, the SHAP value was found to affect obstructive CAD prediction in the following order: troponin T, HbA1c, triglyceride, creatinine clearance, and HDL cholesterol. This means that the SHAP values of HbA1c, HDL cholesterol, triglyceride, and creatinine clearance, which reflect the current state of the disease, were higher than the SHAP values of DM, dyslipidemia, and CKD. Therefore, it may be more helpful in predicting the disease. In our study, even if troponin T was very finely detected within the normal range, it contributed to the prediction of obstructive CAD. Previous studies have reported that elevated levels of troponin T are associated with increased coronary artery plaque volume, structural heart disease, and cardiovascular events (22, 23). Therefore, an ML-based model that incorporates these variables could be more accurate in predicting the disease. Moreover, laboratory data and multiple biomarkers can be directly sampled in an outpatient clinic, and results can be easily obtained; therefore, it is expected that ML algorithms developed based on these data can serve as a pre-test probability model in real-world practice.

The application of the new pre-test probabilities has important consequences in selecting appropriate diagnostic testing. ML-based models may be helpful in clinical decisions when non-invasive diagnostic tests are not available. Furthermore, AI-based integrated analysis of all data, including non-invasive diagnostic tests, will contribute significantly to patients’ precise diagnosis.

This study had several limitations. First, this was a retrospective single-center analysis and thus susceptible to data selection and measurement biases. Second, our ML-based models were not externally validated. Our models were independently divided into training and validation sets to limit overfitting to some extent. In the future, we should conduct a performance test using completely separated test data, which are not used for model development. Third, some values were missing from the data. Missing values could be handled in the boosting algorithm model as the “not available” category. Still, our results were consistent with those obtained with or without missing data imputation (Supplementary Table 2). In the future, detailed and complete hospital-level patient data with minimal missing values will be needed. Fourth, our study did not compare the ML-based model with other non-invasive diagnostic tests. Further randomized control trials comparing the AI-based prediction model and the existing non-invasive stress test are needed to clarify performance power.

## Conclusion

In conclusion, we developed and validated a new prediction model for stable obstructive CAD using ML algorithms. Our ML-based model predicted the probability of obstructive CAD more accurately than the existing pre-test probability of CAD scores. It would be useful to predict the risk of CAD, and helpful to select appropriate non-invasive testing for ischemia.

## Data Availability Statement

The raw data supporting the conclusions of this article will be made available by the authors, without undue reservation.

## Ethics statement

The studies involving human participants were reviewed and approved by the Institutional Review Board of Dankook University Hospital. Written informed consent for participation was not required for this study in accordance with the national legislation and the institutional requirements.

## Author contributions

JK, SL, and SC designed the study. JR and YC assisted in data acquisition and interpretation. BC and WL performed the statistical analyses. DK, S-HL, B-EP, and M-YL contributed to the discussion. JK and TK drafted the manuscript. SL and SC revised the manuscript. All authors read and approved the final version of the manuscript.

## Conflict of Interest

BC and WL were employed by CNAI. The remaining authors declare that the research was conducted in the absence of any commercial or financial relationships that could be construed as a potential conflict of interest.

## Publisher’s Note

All claims expressed in this article are solely those of the authors and do not necessarily represent those of their affiliated organizations, or those of the publisher, the editors and the reviewers. Any product that may be evaluated in this article, or claim that may be made by its manufacturer, is not guaranteed or endorsed by the publisher.

## Abbreviations

AI: artificial intelligence
AMI: acute myocardial infarction
AUROC: area under the receiver operating characteristics
CAD: coronary artery disease
CI: confidence interval
CKD: chronic kidney disease
DM: diabetes mellitus
HbA1c: hemoglobin A1c
HDL: high-density lipoprotein
lightGBM: Light Gradient Boosting Machine
MCC: Matthews correlation coefficient
ML: machine learning
MLP: MulitLayer Perceptron
PCI: percutaneous coronary intervention
SD: standard deviation
SHAP: SHapley Additive exPlanations
SVM: support vector machine
Troponin T: high-sensitivity cardiac troponin T
XG: Extreme gradient.
