# Supplementary material for: Machine learning models of clinically relevant biomarkers for the prediction of stable obstructive coronary artery disease
Source: Front Cardiovasc Med. 2022 Jul 19;9:933803. doi: 10.3389/fcvm.2022.933803 (PMC9343708; doi:10.3389/fcvm.2022.933803)
Supplement: Supplementary file 1 [file Data_Sheet_1.pdf]

Supplementary Material

Supplementary Table S1. Hyperparameters of Machine learning models

Supplementary Table S2. The classification performance in data with missing value imputation

Supplementary Figure S1. Feature importance ranking of all available variables

Supplementary Material

Supplementary Table 1. Hyperparameters of Machine learning models

	Hyper-parameter space	Best Combination of Hyperparameters
**CatBoost**	{'objective': ['CrossEntropy', 'LogLoss'], 'colsample_bylevel': 0.01~0.1, 'depth': [1, 2, 3, 4, 5, 6, 7, 8, 9, 10, 11, 12], 'boosting_type':['Plain', 'Ordered'], 'bootstrap_type': ['Bernoulli', 'MVS']}	{'objective': 'CrossEntropy', 'colsample_bylevel': 0.07204955354411087, 'depth': 5, 'boosting_type': 'Ordered', 'bootstrap_type': 'Bernoulli', 'subsample': 0.3304637109012857}
**XGBoost**	{'booster': ["gbtree", "dart"], 'max_depth': [1, 2, 3, 4, 5, 6, 7, 8, 9], 'eta': 1e-8~1.0, 'gamma': 1e-8~1.0, 'grow_policy': ["depthwise", "lossguide"], 'min_child_weight', 1e-8~7, 'subsample': 0.5~1.0, 'colsample_bytree': 0.5~1.0, 'lambda': 1e-8~1.0, 'alpha': 1e-8~1.0}	{'lambda': 0.37927981109329206, 'alpha': 0.9805763194814385, 'num_boost_round': 37, 'max_depth': 9, 'eta': 0.0014379045325768336, 'gamma': 0.18406078011313776, 'grow_policy': 'lossguide', 'min_child_weight': 5.267513277099358, 'subsample': 0.6856863780740691, 'colsample_bytree': 0.688088947036968}
**LightGBM**	{'objective': ['regression', 'regression_l1', 'huber', 'fair', 'poisson', 'quantile', 'mape', 'gamma', 'tweedie', 'binary'], 'boosting_type': ['gbdt', 'rf', 'dart', 'goss'], 'feature_pre_filter': [True, False], 'lambda_l1': 0~1, 'lambda_l2': 0~1, 'num_leaves': 2~131072, 'feature_fraction': 0~1, 'bagging_fraction': 0~1, 'bagging_freq': 0~10, 'min_child_samples': 0~100}	{'objective': 'binary', 'metric': 'auc', 'verbosity': -1, 'boosting_type': 'gbdt', 'feature_pre_filter': False, 'lambda_l1': 6.0139982784366754, 'lambda_l2': 2.0015289857179124e-07, 'num_leaves': 6, 'feature_fraction': 0.8, 'bagging_fraction': 1.0, 'bagging_freq': 0, 'min_child_samples': 10}
**Gradient Boost**	{'loss': ['deviance', 'exponential'], 'learning_rate': 1e-8~1, 'n_estimators': 100~5000, 'subsample': 0.5~1, 'criterion': ['squared_error', 'friedman_mse', 'mse', 'mae], 'min_samples_split': 2~20, 'min_samples_leaf': 1~20, 'max_depth': 2~15}	{'loss': 'deviance', 'learning_rate': 0.005001710593960871, 'n_estimators': 1900, 'subsample': 0.6346381848474243, 'criterion': 'squared_error', 'min_samples_split': 11, 'min_samples_leaf': 14, 'max_depth': 4}
**Linear SVM**	{'penalty': ['l1', 'l2'], 'loss': ['hinge', 'squared_hinge']}	{'penalty': 'l2', 'loss': 'squared_hinge'}
**KNN**	{'n_neighbors': [5, 10, 15], 'weights': ['uniform', 'distance']}	{'n_neighbors': 10, 'weights': 'distance'}
**GBC**	{'n_estimators': 100~3000, 'criterion': ['gini', 'entropy'], 'max_depth': 4~50, 'min_samples_split': 2~150, 'min_samples_leaf': 1~60, 'max_samples': 0.5~1}	{'n_estimators': 1200, 'criterion': 'gini', 'max_depth': 15, 'min_samples_split': 12, 'min_samples_leaf': 2, 'max_samples': 0.980910966889412}

**MLP**	{‘use_dropout’: [True, False],<br>‘hidden_layer_sizes’: [(58,), (116,), (174,), (232,)],<br>‘n_layers’: [1, 2, 3, 4, 5]}	{‘use_dropout’: True,<br>‘hidden_layer_sizes’: (174,),<br>‘n_layers’: 2}

Supplementary Table S2. The classification performance in data with missing value imputation

Models	AUROC	MCC	Accuracy	Sensitivity	Specificity	PPV	NPV	F1
CatBoost	**0.791**	**0.428**	**0.739**	**0.783**	0.652	**0.815**	**0.606**	**0.799**
XGBoost	0.779	0.413	0.728	0.761	**0.663**	**0.815**	0.587	0.787
LightGBM	0.773	0.369	0.706	0.739	0.641	0.801	0.557	0.769

The bold values indicate the best performance of the 11 models.

AUROC = area under the receiver operating characteristics; GBM = gradient boosting machine; MCC = Matthews correlation coefficients; NPV = negative predictive value; PPV = positive predictive value; XG = extreme gradient.

Supplementary Figure S1. Feature importance ranking of all available variables.

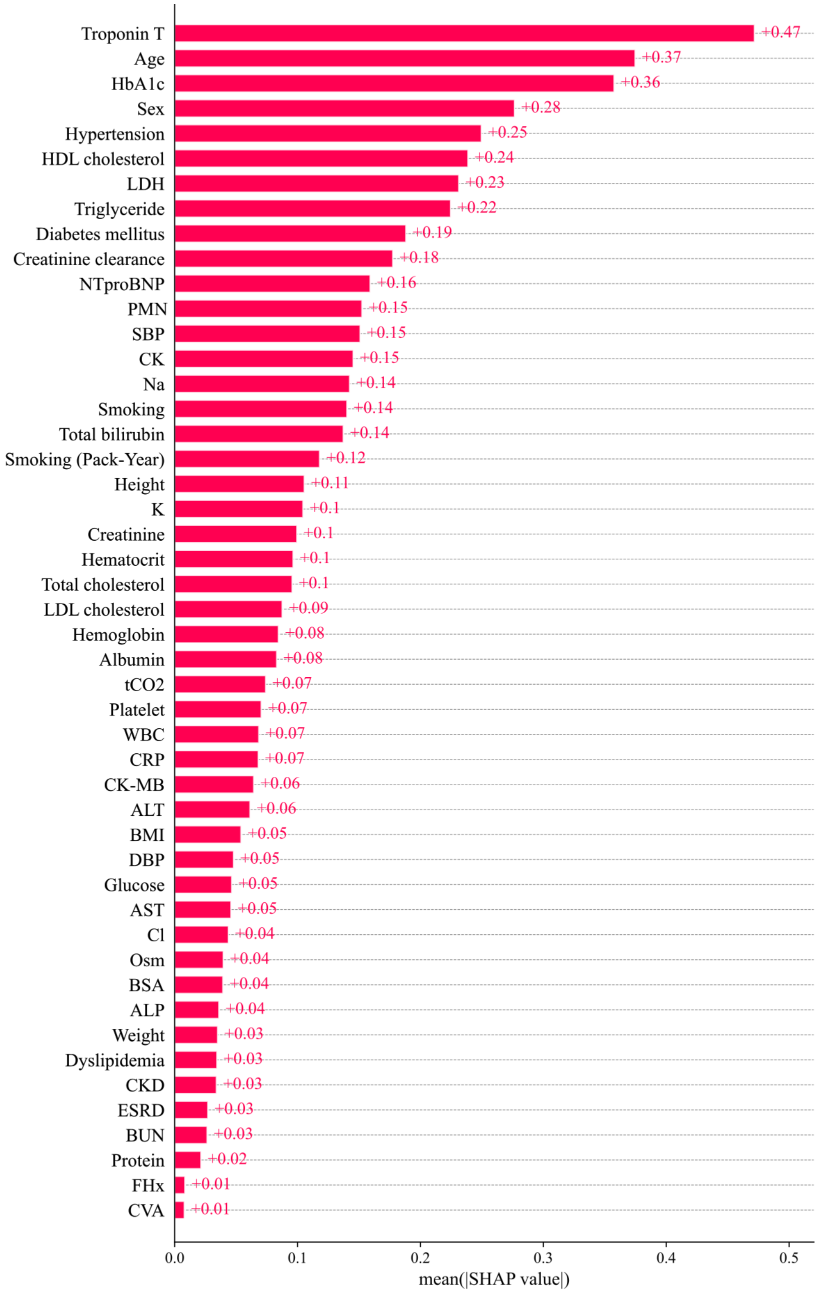

ALP = alkaline phosphatase; ALT = alanine aminotransferase; AST = aspartate aminotransferase; BMI = body mass index; BSA = body surface area; BUN = blood urea nitrogen; CK = creatine kinase; CK-MB = creatine kinase MB isoenzyme; CKD = chronic kidney disease; CRP = C-reactive protein; CVA = cerebrovascular accident; DBP = diastolic blood pressure; ESRD = end-stage renal disease; FHx = family history of atherosclerotic cardiovascular disease; HbA1c = hemoglobin A1c; HDL = high-density lipoprotein; LDH = lactate dehydrogenase; LDL = low-density lipoprotein; Osm = serum Osmolality; PMN = polymorphonuclear leukocyte; SBP = systolic blood pressure; SHAP = SHapley Additive exPlanations; tCO2 = Total CO2; Troponin T = high-sensitivity cardiac troponin T; WBC = White Blood cell
